# Precision Genetic Therapies: Balancing Risk and Benefit in Patients with Heart Failure

**DOI:** 10.1007/s11886-024-02096-5

**Published:** 2024-08-07

**Authors:** Jamie R. Johnston, Eric D. Adler

**Affiliations:** 1 Department of Biomedical Sciences, College of Medicine, Florida State University, Tallahassee, FL 32306 USA; 2 Division of Cardiology, Department of Internal Medicine, University of California San Diego, La Jolla, CA 92037 USA

**Keywords:** Heart Failure, Cardiomyopathy, Muscular Dystrophy, Gene Therapy, Gene Editing, Gene Transfer

## Abstract

**Purpose of Review:**

Precision genetic medicine is evolving at a rapid pace and bears significant implications for clinical cardiology. Herein, we discuss the latest advancements and emerging strategies in gene therapy for cardiomyopathy and heart failure.

**Recent Findings:**

Elucidating the genetic architecture of heart failure has paved the way for precision therapies in cardiovascular medicine. Recent preclinical studies and early-phase clinical trials have demonstrated encouraging results that support the development of gene therapies for heart failure arising from a variety of etiologies. In addition to the discovery of new therapeutic targets, innovative delivery platforms are being leveraged to improve the safety and efficacy of cardiac gene therapies.

**Summary:**

Precision genetic therapy represents a potentially safe and effective approach for improving outcomes in patients with heart failure. It holds promise for radically transforming the treatment paradigm for heart failure by directly targeting the underlying etiology. As this new generation of cardiovascular medicines progress to the clinic, it is especially important to carefully evaluate the benefits and risks for patients.

## Introduction

Heart failure (HF) is a global pandemic that remains a formidable challenge in modern cardiovascular medicine. It is estimated that HF affects at least approximately 64 million people across the globe [1]. While the incidence of HF appears to have stabilized, the prevalence is on the rise because of an aging population in addition to increased availability and use of evidence-based treatments that are prolonging patient survival [2]. While these interventions have increased survival and quality of life in subsets of patients, HF continues to represent a substantial social and economic burden. Gene therapy, defined as the use of genetic material for clinical applications to prevent, treat, or cure human disease, has emerged as a promising strategy to combat this complex clinical syndrome [3]. In contrast to cotemporary therapeutic modalities for HF, gene therapy has the potential to reshape the treatment paradigm by directly targeting the underlying etiology [4].

In this Review article, we summarize recent progress in the field and innovative strategies on the horizon in gene therapy for HF. We begin by describing the key categories of gene therapy, including gene transfer, genome editing, and epigenome editing, along with their respective mechanisms. For recent advances on the rapidly expanding application of RNA interference in cardiovascular precision medicine, we refer the readers to the following excellent review articles [5–7]. We then outline the various gene therapy delivery modalities, including viral and non-viral-mediated approaches. We conclude by discussing the application of gene therapy in the context of rare and common determinants of HF, with a focus on muscular dystrophies and inherited cardiomyopathies. As many of these potential therapies are at preclinical and clinical stages of development, we place particular emphasis on the risks and benefits of these next generation cardiovascular precision medicines.

### Gene Transfer

Gene transfer, otherwise referred to as gene replacement, seeks to deliver a functional copy of a gene to cells or tissues of interest. This is accomplished by designing a viral construct containing the coding sequence for the gene of interest (GOI) and cis-regulatory elements (i.e., gene promoters, enhancers, silencers, microRNA cassettes). Although plasmids can also be used to deliver transgenes, there has been little success with this method for in vivo cardiovascular applications. Selecting the appropriate cis-regulatory elements is critical for restricting transgene expression in the tissue(s) of interest, ensuring that expression levels are optimal for therapeutic efficacy, and for reducing potential cellular toxicity associated with transgene overexpression. Commonly used transgene promoters for muscle gene therapy include desmin (DES), myosin light chain (MLC2v), skeletal muscle alpha actin-based (HSA), muscle creatine kinase and/or myosin heavy chain-based (CK6, CK8, MHCK7), cardiac troponin T (TNNT2), ubiquitous promoters (CMV, CAG), and synthetic derivatives [8]. The length of the GOI is another relevant consideration owing to the size limitation of viral-based gene delivery platforms. For example, adeno-associated virus (AAV) is limited to transgene lengths of approximately 4.7 kb, which precludes the incorporation of larger genes associated with disease [9].

Gene replacement therapy is expected to be most beneficial in the setting of pathogenic loss-of-function (LoF) variants. Nonsense, splice site, and frameshift variants are among the most common LoF variants in genetically determined cardiomyopathy and HF [10]. It is thought that these mutations generate truncated, non-functional proteins. However, the activity of nonsense mediated messenger RNA (mRNA) decay may prevent translation of these mutant proteins in the cell and instead generate a null allele [11]. Additionally, expressing the functional copy of gene may have salutary effects in the context of certain LoF missense variants associated with sarcomeric cardiomyopathy, which we discuss further in a later section of this article.

### Somatic Genome Editing

Therapeutic genome editing involves modifying the endogenous deoxyribonucleic acid (DNA) sequence in somatic cells. In contrast to gene transfer, genome editing has the potential to permanently correct LoF and gain-of-function (GoF) pathogenic variants associated with HF and cardiomyopathy. Clustered regulatory interspaced palindromic repeats (CRISPR)-Cas9 is an immune defense system in bacteria that has been repurposed for eukaryotic genome editing in biomedical research [12]. Genome editing with CRISPR-Cas9 requires two fundamental components: a single guide RNA that specifically targets a DNA region of interest and a modified RNA-guided endonuclease (Cas9) that induces double-strand DNA breaks [13]. Following DNA repair by endogenous cellular machinery (homology directed repair in dividing cells or non-homologous end joining), gene knock-in or knockout is achieved [14]. The CRISPR-Cas9 system can edit the genome through targeted conversion, insertion, or deletion of DNA base pairs.

Further engineering the CRISPR-Cas9 has led to the development of base and prime editors, which can install targeted point mutations in the genome with higher efficiency compared to the conventional system [15, 16]. Base and prime editing does not rely on double-strand DNA breaks, occurs in the absence of a donor DNA template, and is active in non-dividing cells. This approach may be particularly beneficial for editing genomic DNA in the post-mitotic contractile cells of the heart and skeletal muscle [17]. While CRISPR-based therapeutic genome editing offers several advantages over gene replacement therapy, it has a few important drawbacks. Despite its relatively high precision in genome editing, there is a major concern for off-target activity and oncogenic potential. It is especially critical to ensure germline DNA, which is transmitted to offspring, is not edited in the process. There is accumulating evidence of host immune responses to the bacterial endonucleases [18–20]. Not only could this ultimately reduce the effectiveness of in vivo genome editing, but it may also cause host cytotoxicity and serious side effects in patients. Current CRISPR-based gene editing strategies also require the design and development of mutation-specific therapies, which is a lengthy and costly process.

### Epigenome Editing

The CRISPR-Cas9 system has been further engineered to modulate the expression of endogenous genes without the requirement for DNA damage. This is accomplished by fusing transcriptional or epigenetic effector proteins to a catalytically inactive Cas9 (dCas9) and targeting the machinery to a DNA region of interest [21, 22]. In theory, this could be an effective approach to modulate the expression of genetic modifiers in the setting of disease. While RNA-guided dCas9 is capable of moderate gene repression without effector proteins by blocking RNA polymerase/transcription factor binding, direct fusion with Krüppel-associated box (KRAB) or four concatenated mSin3 interaction domains (SID4X) enables robust downregulation of endogenous gene expression [17]. In contrast, fusion of VP64, p65, or VPR activation domains to dCas9 enhances endogenous gene transcription [17].

Endogenous gene expression can also be manipulated by fusing dCas9 to effector proteins that modify epigenomic markers. For example, fusion of dCas9 to the catalytic domain of DNA (cytosine-5)-methyltransferase 3A (DNMT3A) has been shown to promote site-specific DNA methylation and subsequent repression of proximal genes [23, 24]. Fusion of dCas9 with the catalytic core of histone acetyltransferase p300 was shown to increase histone H3 lysine 27 acetylation, a marker of active gene enhancers, and up-regulate the expression of nearby target genes [25]. Alternatively, fusion of dCas9 with lysine-specific histone demethylase 1 (LSD1) has been shown to repress targeted gene expression by altering the levels of specific epigenome modifications at a nearby enhancer region [26]. In contrast to catalytically active Cas9 systems, a key advantage of potential therapies leveraging CRISPR-dCas9 is the ability to modulate gene expression in vivo without carrying the risks associated with directly altering the genetic code.

### Messenger RNA Technologies

The COIVD-19 pandemic catalyzed the widespread development and implementation of mRNA technologies to combat disease. Messenger RNA medicines, which can be thought of programmable biological software, involve the synthesis and modification of mRNA molecules that encode proteins that stimulate immune response against infectious agents or that correct genetic defects underlying various diseases [27]. Owing to its versatility and relative ease of manufacturing, this innovative technology has the potential to revolutionize medical therapy across a wide spectrum of ailments. Although in early stages, mRNA-based medicines are also extending to therapeutic applications for cardiovascular disease [28]. In a phase 2a clinical trial, surgeons administered naked vascular endothelial growth factor (VEGF) mRNA via intramyocardial injection at thirty sites within ischemic regions in patients undergoing coronary artery bypass grafting (CABG) [29]. Subsequent analysis revealed decreased plasma brain natriuretic peptide (BNP) levels and increased walking distance on the treadmill, suggesting potential beneficial effects on cardiovascular function. While these results are encouraging, this approach may be further optimized by refining the delivery method and/or enhancing mRNA stability to durably reverse myocardial ischemia. Fibrosis is a pathological hallmark of heart disease and plays a key role in progression to heart failure. In 2022, an mRNA-based approach was evaluated for its potential effectiveness in reversing cardiac fibrosis in an in vivo preclinical model of heart injury [30]. In this elegant study, lipid nanoparticles containing mRNA molecules that encode a chimeric antigen receptor (CAR) were used to generate CAR T cells in mice, resulting in a population of therapeutic T lymphocytes that abolished activated fibroblasts, attenuated myocardial fibrosis, and restored cardiac function following injury. There is also an ongoing Phase 1 clinical trial by Moderna to evaluate the safety and tolerability an investigational mRNA-based therapeutic (mRNA-0184) for the treatment of heart failure (NCT05659264). Messenger RNA-0184 is an intravenously administered lipid nanoparticle formulation that contains modified mRNA encoding a human relaxin-2 fusion protein. Relaxin-2 is a naturally occurring hormone that has been shown to exert beneficial effects on cardiovascular remodeling and renal function. Together, these studies highlight the tremendous potential of harnessing mRNA technology to treat cardiovascular disease arising from diverse etiologies.

### Delivery Modalities

Achieving efficient and targeted delivery of genetic medicines to the heart has historically represented a major hurdle thwarting the development of precision therapies for HF [31]. However, recent progress in viral- and non-viral-mediated gene delivery modalities is enabling unprecedented access to the cardiovascular system in a targeted manner.

#### Adenoviruses

Adenoviruses are non-enveloped viruses that are comprised of an icosahedral capsid [32]. The adenoviruses genome is 54 kilobases and is composed of double-stranded DNA. Adenoviruses have a few advantages over AAVs, such as the capacity to package larger transgenes and achieve higher rates of viral transduction. However, adenoviruses appear to be substantially more immunogenic and transgene expression is limited to a transient period [33]. Furthermore, administration of a therapeutic adenovirus led to the death of a patient with ornithine transcarbamylase deficiency due to toxicity associated with a systemic inflammatory response [34]. Consequently, momentum for developing gene therapy adenoviral vectors waned, and attention instead has focused on adeno-associated viruses (AAVs).

#### Adeno-Associated Viruses

Similar to adenoviruses, AAVs are non-enveloped vectors with an icosahedral capsid. However, in contrast to adenoviruses, AAVs contain a smaller (~ 4.7 kilobase) DNA genome that is single stranded [32]. Recombinant AAVs used for therapeutic purposes are rendered replication deficient and persist as non-integrating, extrachromosomal structures in the host cell, thereby providing stable transgene expression. While AAVs appear to display a lower immunogenic profile compared to adenoviruses and lentiviruses, it is not negligible. AAV has been shown to trigger the innate and adaptive immune systems accompanied by inflammation in the host [35–37]. The immunogenicity of AAVs can culminate in toxicity and reduced biologic effects. Furthermore, neutralizing anti-AAV antibodies are relatively prevalent in the general population and can cross-react between different serotypes [38]. Nevertheless, AAVs hold tremendous promise in serving as safe and effective delivery vehicles for precision genetic medicines in the setting of HF [39].

While naturally occurring serotypes like AAV9 exhibit relatively high cardiac and skeletal muscle tropism, recent advancements in AAV capsid engineering have led to the development of AAVs demonstrating unprecedented transduction efficiencies in striated muscle. Using directed evolution of AAV capsids, two independent groups recently reported the development of these next generation AAV variants dubbed “AAVMYO” and “MyoAAV” [40, 41] In murine and primates preclinical studies these myotropic capsids has enabled efficient delivery of therapeutic transgenes at significantly lower doses—up to 250 times lower than what is necessary for naturally occurring AAVs [42]. Advancements in capsid engineering such as these are critical to improving efficacy and lowering the cost of good for potential gene therapies.

#### Lentiviruses

Lentiviruses are enveloped, single-stranded RNA vectors that can be harnessed for gene therapy applications. They are capable of efficiently transducing dividing and post-mitotic cells and can accommodate transgenes up to approximately 9 kilobases in size [32]. However, a major limitation is the potential risk for insertional mutagenesis, as lentiviral vectors randomly integrate into coding regions of the host genome to induce long term transgene expression [43, 44]. While this risk may be minimal in terminally differentiated cells of the heart and skeletal muscle, it is not zero. Self-inactivating lentiviral vectors have been developed to further minimize this risk [45], but with availability of safer alternatives such as AAVs, we are not aware of any current reports on lentiviruses being developed for in vivo cardiovascular gene therapy.

#### Non-Viral

To circumvent the concern of immunogenicity and limited packaging size associated with viral-based gene delivery vehicles, non-viral vectors have been developed for use in gene therapy. Non-viral vectors include inorganic nanoparticles, exosomes, cationic polymers, and lipid nanoparticles, as well as combinations thereof [46]. In contrast to existing virus-based modalities, non-viral vectors tend to be easier to scale up for mass production. To confer tissue specificity of non-viral vectors, specific targeting ligands are conjugated to the surface of the particles [47]. This is a rapidly expanding area of research and development, especially for RNA-based genetic medicines [48]. Indeed, mRNA-based vaccines for the COVID-19 pandemic were formulated with lipid nanoparticles and were clinically proven to be relatively safe and effective [49]. Recent studies have shown encouraging results for in vivo delivery of gene editing machinery using lipid nanoparticles to treat disorders of the liver, including amyloid disease and hypercholesterolemia. [50, 51] As the field makes additional progress in the future, non-viral vectors may have increased utility for cardiovascular gene therapy. Current limitations include the inability to effectively deliver RNA therapeutics to striated muscle.

### Targets for Cardiovascular Gene Therapy

HF is a complex clinical syndrome influenced by the interplay of genetic and environmental factors [4]. Genetic contributions can be subdivided into monogenic and polygenic cardiac disorders [10]. Based on therapeutic tractability and unmet need, cardiomyopathies and muscular dystrophies comprise most of the current targets for HF gene therapy, which we outline below.

#### Barth Syndrome

Barth syndrome is a rare and potentially lethal disorder characterized by cardiomyopathy, skeletal muscle weakness, neutropenia, organic aciduria, growth delay, and exercise intolerance [52]. It affects approximately 1 in 0.3 to 0.4 million live births and primarily males [53]. It is caused by pathogenic mutations in the Tafazzin gene (*TAZ*), which is located on the X chromosome, and encodes a protein (TAZ) located in the inner mitochondrial membrane [52]. TAZ is an enzyme essential for the biogenesis of cardiolipin, a phospholipid important for maintaining mitochondrial structure and lipid metabolism [54]. Cardiomyopathy and neutropenia represent the leading causes of mortality in patients with Barth syndrome, and there are no targeted therapies currently available [55]. A preclinical study recently tested the efficacy of an AAV-based *TAZ* gene transfer therapy in a knock-out mouse model of Barth syndrome [56]. Neonatal *Taz* knock-out mice that received high-dose AAV9 displayed significantly increased survival compared to untreated *Taz* knock-out mice. Furthermore, the treated mice showed protection against cardiac dysfunction and reduced myocardial fibrosis. Results from additional experiments suggested that increased survival was primarily due to TAZ protein replacement in skeletal muscle. Based on this proof-of-concept study, gene transfer may be a viable approach for treating Barth syndrome.

#### Danon Disease

Danon disease is a rare X-linked lysosomal storage disorder characterized by progressive hypertrophic cardiomyopathy (HCM), skeletal muscle weakness, liver pathology, and cognitive disability [57]. It is a monogenic disease caused by loss-of-function mutations in the lysosomal-associated membrane protein 2 gene (*LAMP2*) [58]. LAMP-2 protein deficiency leads to impaired autophagic flux and subsequent accumulation of autophagic vacuoles in tissues [59]. While the exact prevalence is uncertain, it has been reported that 1–4% of patients with hypertrophic cardiomyopathy have a *LAMP2* variant, necessitating an unmet need to develop precision therapies [60, 61]. In 2020, a preclinical study evaluated the efficacy of an AAV9-based *LAMP2B* gene transfer therapy in a *Lamp2b* knock-out mouse model of Danon disease [62]. Adult and adolescent knock-out mice treated with systemic administration of AAV9-*LAMP2B* demonstrated increased survival, cardiac function, and autophagic flux and reduced transaminases compared to untreated control mice. This proof-of-concept study paved the way for the development of an AAV9-based human gene therapy vector by (RP-A501) that is currently being tested in a phase 1 clinical trials (NCT03882437), with plans to begin phase 2 clinical trials by the end of 2023.

#### Sarcomeric Cardiomyopathy

Autosomal dominant mutations in the genes encoding sarcomeric proteins, the contractile regulatory proteins in striated muscle cells, are a leading cause of inherited cardiomyopathies, including HCM, dilated cardiomyopathy (DCM), and restrictive cardiomyopathy (RCM) [63]. The genes strongly associated with sarcomeric HCM are myosin heavy chain (*MYH7*) and myosin binding protein C (*MYBPC3*), whereas mutations in the titin gene (*TTN*) represent the leading cause of sarcomeric DCM. Mutations in the genes encoding thin filament proteins, including troponin subunits (*TNNI3*, *TNNT2*, *TNNC1*) and alpha tropomyosin (*TPM1*) are also tightly associated with genetic cardiomyopathy [64]. In two recent studies reported by independent groups, CRISPR-Cas9 adenine base editing was successfully used to prevent the development and progression of HCM in well-established mouse models [65, 66]. In another study using a *Mybpc3* knock out mouse model of HCM, single dose administration with AAV9-*MYBPC3* gene transfer in neonatal mice was sufficient to prevent the development of HCM [67]. This proof-of-concept study paved the way for the development of an AAV9-*MBYPC3* gene transfer therapy vector (TN-201) that is currently being tested in a phase 1 clinical trial to assess safety and efficacy (NCT05112237). AAV gene transfer may be effective for treating certain missense mutations linked to cardiomyopathy. In theory, delivery of an exogenous wild type sarcomeric protein in the setting of a diseased allele could potentially outcompete and stoichiometrically replace the endogenous mutant protein, thereby normalizing contractile function [11]. While additional studies are needed to assess this possibility, it is conceivable that this approach may be viable for treating a large proportion of patients with sarcomeric cardiomyopathy.

#### BAG3 Cardiomyopathy

Mutations in the BCL2-associated athanogene 3 (*BAG3*) gene have been associated with the development of DCM in humans [68]. The majority are truncating or deletion variants, resulting in reduced BAG3 protein levels. Decreased levels of BAG3 protein are also observed in the setting of end-stage HF caused by myocardial infarction [69]. Low levels of BAG3 protein are associated with impaired sarcomere contractility and decreased myofilament protein turnover [70]. Evidence from several preclinical studies supports the potential use of gene therapy to treat cardiomyopathy and failure [71]. For example, AAV9-mediated gene transfer of BAG3 in haplo-insufficient mice suggested beneficial effects on contractile function [72]. In another study, mice with HF secondary to experimental myocardial infarction were treated with AAV9-*BAG3*, leading to increased myofilament protein turnover, and restoration of myocardial contractility [73]. More recently, a small study in healthy Yucatan mini pigs revealed that low-dose AAV9-*BAG3* gene transfer by catheter-guided retrograde coronary sinus infusion resulted in widespread myocardial transduction [74], pointing to a potentially viable approach to efficiently deliver *BAG3* transgene in the setting of cardiomyopathy and HF.

#### Duchenne Muscular Dystrophy

Duchenne muscular dystrophy (DMD) is a severe, X-linked recessive neuromuscular disorder caused by mutations in the dystrophin gene (*DMD*) that lead to dystrophin protein deficiency [75]. Dystrophin maintains the structural integrity of the sarcolemma in striated muscle cells of the heart and skeletal muscle. Patients with DMD exhibit progressive skeletal muscle weakness, DCM, and often death by the third decade of life [76]. While the enormous size of the full-length dystrophin coding sequence precludes it from being packaged into a viral vector, innovative strategies have been developed to enable gene therapy for DMD [77]. The creation of mini/micro-dystrophin constructs, which contain only the critical domains of full-length dystrophin, have shown promise for restoring muscle structure and function in the setting of DMD.

In June of 2023, the U.S. Food and Drug Administration (FDA) granted approval of the first AAV gene therapy product (delandistrogene moxeparvovec, tradename ELEVIDYS) for the treatment of pediatric patients, 4 to 5 years of age, with DMD [78]. This decision was based on evidence of increased micro-dystrophin protein expression in treated patients from phase 1/2 clinical trials. ELEVIDYS is a recombinant AAV, serotype rh74, containing codon optimized human micro-dystrophin under the control of the muscle specific MHCK7 promoter [79]. Proof-of-concept studies have also reported the use of AAV-mediated base and prime editing in mouse models of DMD [80–82]. In addition to AAV gene therapy methods, there are 4 currently FDA approved exon-skipping antisense oligonucleotide (ASO) therapies for DMD [83], although these are mutation specific and require repeated dosing. Furthermore, it is unclear whether exon-skipping strategies improve myocardial function.

#### Fabry Disease

Fabry disease is an X-linked lysosomal storage disorder caused by mutations in the alpha galactosidase A gene (*GLA*) [84]. These mutations typically reduce the enzymatic activity of alpha galactosidase A protein, resulting in lysosomal accumulation of glycosphingolipid substrates such as cerebroside trihexosides. Fabry disease affects multiple organ systems, frequently culminating in renal failure, severe heart disease, cerebrovascular events, and premature death [85, 86]. There are several ongoing studies assessing the safety and efficacy of gene therapy for Fabry disease. AVR-RD-01 is an investigational ex vivo gene therapy in which a patient’s own hematopoietic stem cells are genetically modified with a lentivirus to express functional alpha galactosidase A and then transplanted back into the patient (NCT03454893). Unfortunately, patient enrollment for this study was terminated owing to poor grafting in 5 participants in the open-label Phase 2 clinical trial. ST-920 (isaralgagene civaparvovec) is a single-dose AAV gene transfer investigational therapy currently in Phase 1/2 clinical trials to assess safety and tolerability in subjects with Fabry disease (NCT04046224). It is a recombinant AAV2/6 vector containing the full length human *GLA* coding sequence. Preliminary results from the trial suggest a favorable impact on progression of neuropathy in Fabry patients, and the developers intend to move forward with Phase 3 clinical trials by the end of 2023. 4D-310 is another single-dose intravenous AAV-based investigational gene transfer therapy for Fabry disease currently in a Phase 1/2 clinical trials to assess safety, tolerability, and pharmacodynamics (NCT04519749). 4D-310 is comprised of an engineered cardiotropic AAV vector (C102) and transgene cassette containing codon-optimized full-length human *GLA* driven by the CAG promoter. In February 2023, the study was placed on hold by the FDA due to reports of atypical hemolytic uremic syndrome in 3 of the 6 participants who received the treatment. In September of 2023, a pre-clinical study evaluated an AAV2/8 human *GLA* gene transfer approach in a mouse model of Fabry disease [87]. Upon systemic administration, the treated mice showed increased GLA enzyme activity in plasma and relevant tissues, which was maintained for up to 38 weeks. These findings further support the development of AAV-based precision gene therapies for Fabry disease.

#### Transthyretin Amyloid Cardiomyopathy

Transthyretin amyloid cardiomyopathy (ATTR-CM) is a disorder characterized by extracellular deposition of misfolded transthyretin protein that accumulates primarily in the myocardium and peripheral nerves [88]. It can be caused by pathogenic variants in the *TTR* gene, referred to as hereditary ATTR-CM, or occur as an age-related phenomenon, referred to as wild-type ATTR-CM. Both forms of ATTR-CM can lead to severe HF [89]. Although small interfering RNA (siRNA) and ASO-based medicines are currently available on the market, these therapies require long-term administration to sustain benefit [90]. A new in vivo CRISPR-based gene editing therapy (NTLA-2001) is currently being investigated for the treatment of ATTR-CM (NCT04601051). NTLA-2001 is delivered by a proprietary lipid nanoparticle formulation that is engineered to be internalized by the low-density lipoprotein receptor on hepatocytes, CRISPR-Cas9 editing induces a frameshift mutation in the *TTR* gene, leading to decreased transthyretin protein production. Preclinical studies in rodent models and nonhuman primates demonstrated that a single intravenous infusion of NTLA-2001 led to reductions in transthyretin levels that persisted over at least a 12-month period [91, 92]. In August 2021, interim results reported that NTLA-2001 appears to be effective at reducing serum transthyretin levels in humans, with only mild adverse events [92]. In October of 2023, the FDA cleared the Investigational New Drug (IND) application for NTLA-2001, allowing the initiation of a pivotal Phase 3 clinical trials in the U.S. by the end of the year.

#### Limb-Girdle Muscular Dystrophy

Limb-girdle muscular dystrophy (LGMD) represents a group of progressive neuromuscular disorders that lead to profound skeletal muscle weakness and wasting [93]. There are currently no targeted treatments available and, as a monogenic disorder, LGMD is readily amenable to gene therapy. Severe forms of LGMD, such as LGMD type 2E (LGMD2E) are associated with cardiomyopathy and arrhythmias [94, 95]. LGMD2E is caused by mutations in the beta sarcoglycan gene (*SGCB*), leading to beta sarcoglycan deficiency [96]. SRP-9003 (bidridistrogene xeboparvovec) is an investigational gene transfer therapy currently being tested in Phase 1/2 clinical trials for LGDM2E (NCT03652259). SRP-9003 uses AAVrh74 vector and is designed to deliver a functional copy of beta sarcoglycan under the control of the muscle-specific MHCK7 promoter [97]. Recent preclinical studies in mice have also shown promise for using AAV-based gene transfer therapy to treat LGMD type 2C/R5, which is caused by mutations in the gamma sarcoglycan gene (*SGCG*) that lead to protein deficiency [98]. Restoration of gamma sarcoglycan gene and protein expression was observed in skeletal muscles and heart. Improvements in skeletal muscle function were also observed in the treated mice. These results further support the concept of using gene therapies for muscular dystrophies associated with cardiac dysfunction.

#### Hurler Syndrome

Mucopolysaccharidosis type I (Hurler syndrome) is an inherited lysosomal storage disease characterized by hepatosplenomegaly, musculoskeletal abnormalities, retinal degeneration, and cardiomyopathy [99]. Early mortality in patients with Hurler syndrome is typically associated with cardiopulmonary failure. It is caused by mutations in the gene encoding alpha-L-iduronidase (*IDUA*), leading to enzyme deficiency and accumulation of glycosaminoglycan substrates [100]. While several gene-based strategies to treat Hurler syndrome have been previously reported, these approaches are limited by potential loss of the episomal transgene, risk of insertional mutagenesis, and lack of precise targeting [101]. More recently, a new AAV9-based CRISPR adenine base editing therapy was tested in a preclinical mouse model and showed encouraging results [102]. In utero base editing in a mouse model of Hurler syndrome led to long-term correction of the pathogenic mutation in the heart and liver along with improved survival. The treated mice displayed amelioration of pathology in multiple organs including the heart, brain, and liver. Importantly, there were also significant improvements in cardiac and skeletal muscle function, as well as neurobehavioral phenotypes [102].

#### Marfan Syndrome

Marfan syndrome is among the most common inherited disorders of connective tissue [103]. Marfan syndrome is caused by mutations in the fibrillin-1 gene (*FBN1*), which impacts multiple organ systems, including the skeleton, eyes, heart, lungs, and blood vessels. Cardiovascular disease (aortic root dissection, DCM, and HF) accounts for more than 90% of early deaths in patients with Marfan syndrome, necessitating a need for targeted therapies to treat this disorder [104]. A recent proof-of-concept study showed the possibility of using base editing in embryos as an early therapeutic intervention for Marfan syndrome [105]. The results showed a correction efficiency of roughly 89%, with no detection of indels or off-target editing. Therefore, base editing may represent a potential strategy for treating Marfan syndrome.

#### Progeria (Hutchinson-Gilford Syndrome)

Hutchinson-Gilford progeria syndrome (HGPS) is a rare, autosomal dominant premature aging syndrome caused by mutations in the *LMNA* gene [106]. Early death in children with HGPS is associated with accelerated cardiovascular disease, including myocardial infarction and stroke. In a recent proof-of-concept study, adenine base editing was used to correct a HGPS mutation with high efficiency in cultured fibroblasts from patients and in a mouse model of HGPS [107]. In a mouse model of Progeria, a single dose of the base editor delivered by AAV9 led to correction of the pathogenic variant in various organs six months post-injection. Furthermore, the treated mice showed rescue of vascular pathology and significantly improved the median lifespan. This early preclinical study demonstrates the potential power of harnessing precision gene editing to treat HGPS.

#### Friedreich’s *Ataxia*

Friedreich’s ataxia is an autosomal recessive disorder with a prevalence of 1 in 40,000 [108]. It is characterized clinically by progressive neurodegeneration, cerebellar ataxia, cardiomyopathy, and heart failure [108]. At the molecular level, a genetic defect in the frataxin gene (*FXN*) leads to frataxin protein deficiency and mitochondrial dysfunction [109]. There is an ongoing Phase 1 clinical trial to assess the safety and preliminary efficacy of an AAV-based gene transfer approach (AAVrh.10hFXN) to treat Friedreich’s ataxia-associated cardiomyopathy (NCT05302271). This study commenced in February 2022 and the clinical trial is actively recruiting participants. The AAVrh.10hFXN vector is administered intravenously and carries the coding sequence for human *FXN*. In preclinical studies, AAV gene therapy approaches led to correction of cardiomyopathy phenotypes in mouse models of Friedreich’s ataxia [110, 111]. Based on these encouraging proof-of-concept studies, gene transfer may represent a viable therapeutic approach for patients with cardiomyopathy caused by Friedreich’s ataxia [112].

#### Pompe Disease

Pompe disease is an inherited lysosomal storage disorder caused by recessive mutations in the acid alpha glucosidase gene (*GAA*) [113]. These mutations result in alpha glucosidase protein deficiency and excess lysosomal glycogen accumulation in cardiac, skeletal, and smooth muscles. Infantile onset Pompe disease is clinically characterized by severe HCM, hypotonia, and respiratory insufficiency [114]. Preclinical studies have supported the concept of harnessing gene therapy to treat Pompe disease [115]. Without treatment, this disorder is often fatal within 1 year of age, necessitating a need to develop precision therapies. There is an ongoing Phase 1/2 clinical trial to evaluate the safety and efficacy of AT845, an AAV8 vector containing a functional copy of human *GAA* delivered by intravenous infusion, in adult patients with late-onset Pompe disease (NCT04174105). A separate Phase 1 clinical trial is also assessing the safety and bioactivity of ACTUS-101 in patients with late-onset Pompe disease (NCT03533673) [116]. ACTUS-101 is an AAV2/8 vector containing a functional copy of *GAA* for expressing hepatocytes. SPK-3006 is a third investigational gene therapy in Phase 1/2 clinical trials to evaluate the safety, tolerability, and efficacy in patients with late-onset Pompe disease (NCT04093349). SPK-3006 is a recombinant liver-directed AAV vector designed to drive the expression of secretable GAA protein upon a single intravenous infusion. In contrast to enzyme-replacement therapy, which requires repeated administration, these potential AAV precision gene therapies may have the potential to provide long-term for patients with Pompe disease.

## Concluding Remarks

Human gene therapy holds tremendous potential for revolutionary treatments in cardiovascular medicine [117]. As the technologies continue to advance and undergo refinement, opportunities for precise targeting of these devastating disorders will likely expand. Gene transfer and gene editing approaches have unequivocally proven therapeutically effective in preclinical models for heart failure, thereby supporting progression for further evaluation in clinical trials [118]. While some of these investigational therapies have already shown benefit in humans, there remains substantial challenges that necessitate cautious navigation. Safety is the most crucial challenge currently facing the field. The death of a Duchenne muscular dystrophy (DMD) patient in a recent clinical trial using AAV-based gene therapy serves as sobering reminder that minimizing risks of these interventions must occur before widespread clinical implementation [119].

Improving the safety of human gene therapies must be a priority; this will be mandated if the hope of using AAV as an early intervention or for diseases less morbid than DMD. Further research is necessary to better understand the host immune response to the viral vector. The discovery of new capsid variants with increased cardiac tropism may mitigate this risk by significantly lowering the required dose to achieve efficacy. Another issue facing viral-based gene therapies is the potential presence of pre-existing immunity in patients, which could reduce the effectiveness of these drugs and prevent redosing. This problem may be resolved in the future as non-viral delivery such as lipid nanoparticles methods continue to emerge. Immunogenicity is also germane to CRISPR-based therapies, as the Cas9 endonuclease is derived from bacteria.

Preventing transgene expression in unwanted tissues represents another challenge. While the use of tissue-specific promoters and other regulatory elements lowers this risk, it is not zero. The development of synthetic promoters and implementation of novel regulatory elements into therapeutic constructs may provide an avenue for addressing this obstacle. In contrast to gene transfer therapy, gene editing carries the unique risk of off-target mutagenesis, whereby new mutations are introduced at undesired genetic loci. Use of catalytically inactive or higher fidelity Cas proteins have the potential to minimize this risk.

While studies in pre-clinical models suggest durability of gene therapies and absence of negative long-term effects, it is unclear whether this will translate to humans. Meticulous design of clinical trials and close, consistent monitoring is of paramount importance as gene therapies move to the clinical setting. The high cost associated with research, development, and manufacturing of these innovative medicines introduces challenges surrounding affordability and access. Overcoming these myriad challenges requires multidisciplinary collaboration among regulatory agencies, scientists, ethicists, healthcare professionals, and active engagement with patients and families in need.

## Data Availability

No datasets were generated or analysed during the current study.
